# SFG Synthesis of General High-Order All-Pass and All-Pole Current Transfer Functions Using CFTAs

**DOI:** 10.1155/2014/271926

**Published:** 2014-02-11

**Authors:** Worapong Tangsrirat

**Affiliations:** Faculty of Engineering, King Mongkut's Institute of Technology Ladkrabang (KMITL), Ladkrabang, Bangkok 10520, Thailand

## Abstract

An approach of using the signal flow graph (SFG) technique to synthesize general high-order all-pass and all-pole current transfer functions with current follower transconductance amplifiers (CFTAs) and grounded capacitors has been presented. For general *n*th-order systems, the realized all-pass structure contains at most *n* + 1 CFTAs and *n* grounded capacitors, while the all-pole lowpass circuit requires only *n* CFTAs and *n* grounded capacitors. The resulting circuits obtained from the synthesis procedure are resistor-less structures and especially suitable for integration. They also exhibit low-input and high-output impedances and also convenient electronic controllability through the *g*_*m*_-value of the CFTA. Simulation results using real transistor model parameters ALA400 are also included to confirm the theory.

## 1. Introduction and Motivation

In 2008, the conception of the current follower transconductance amplifier (CFTA) has been introduced [1]. The CFTA device is slightly modified from the conventional current differencing transconductance amplifier (CDTA) [2] by replacing the current differencing unit with a current follower. Conceptually, the CFTA element is a combination of the current follower and the multioutput operational transconductance amplifier. Consequently, several structures for realizing current-mode active filters using CFTAs were developed [3–7]. Interesting circuit realizations of general *n*th-order current-mode all-pass filters can be found in [8–15]. However, the works in [8–13] do not include electronic controllability. Moreover, filter structures in [8–12, 15] needed external passive resistors. Although the electronically tunable filters were reported in [14, 15], the input terminals of these structures are not in low-impedance level. The configuration of [15] also employs an external passive resistor for CDTA-based circuit realization. In addition, the realization of general high-order all-pole lowpass current transfer function using current conveyors was introduced in [16]. The circuit still requires a large number of active and passive components, at most *n* + 1 current conveyors, *n* resistors and *n* capacitors.

In this paper, the signal flow graph (SFG) procedure is applied to synthesize the high-order, all-pass, and all-pole current transfer functions using CFTAs as active elements together with grounded capacitors as passive elements. The approach is based on drawing a signal flow graph directly from the given transfer function and then obtaining, from the graph, the active-*C* filter involving CFTAs. The design procedure shows that the resulting structures are canonical in the number of active components, *n* + 1 CFTAs for realizing *n*th-order all-pass circuit and *n* CFTAs for realizing *n*th-order all-pole circuit. Only *n* grounded capacitors are used as passive components for all circuit realizations, making them suitable for integration point of view. As desired, the realized circuits have low-input and high-output impedance properties, which especially enable cascading. The circuits also have low sensitivity characteristics, and exhibit electronic controllability of important filter coefficients via transconductance gains (*g*_*m*_) of CFTAs. To demonstrate the proposed approach, the third-order current-mode all-pass filter and Butterworth all-pole lowpass filter were designed and simulated using PSPICE program.

## 2. Basic Concept of the CFTA

**Figure 1:**
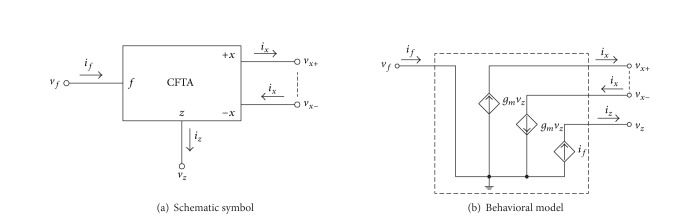
The CFTA.

The symbolic representation of the CFTA and its behavior model are shown in Figure 1. Assuming the standard notation, the terminal defining relations of this device can be characterized by the following [3–6]:

(1) $$\begin{array}{c} v_{f}=0,\;\;i_{z}=i_{f},\;\;i_{x}=g_{m}v_{Z}=g_{m}Z_{Z}i_{Z}, \end{array}$$

where *g*_*m*_ is the transconductance gain of the CFTA and *Z*_*z*_ is an external impedance connected to the *z*-terminal. The CFTA consists essentially of the current follower at the input part and the multioutput transconductance amplifier at the output part. According to (1) and Figure 1, the *f*-terminal forms the current input terminal at ground potential (*v*_*f*_ = 0) and the output current at the *z*-terminal (*i*_*z*_) follows the current (*i*_*f*_) through the *f*-terminal. The voltage drop at the *z*-terminal (*v*_*z*_) is then converted to a current at the *x*-terminal (*i*_*x*_) by a *g*_*m*_-parameter. In general, the *g*_*m*_-value is adjustable over several decades by a supplied bias current/voltage, which lends electronic controllability to design circuit parameters.

**Figure 2:**
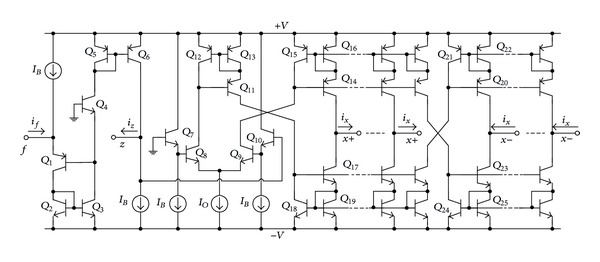
Implementation of the CFTA.

The possible implementation of the bipolar technology-based CFTA is shown in Figure 2 [6, 7]. It is mainly composed of a current follower circuit constructed by transistors *Q*_1_–*Q*_6_ and a multiple-output transconductance amplifier *Q*_7_–*Q*_25_. In this case, the transconductance gain (*g*_*m*_) of the CFTA is directly proportional to the external bias current *I*_*o*_, which is approximately equal to

(2) $$\begin{array}{c} g_{m}=\frac{I_{o}}{2V_{T}}, \end{array}$$

and *V*_*T*_≅26 mV at 27°C.

## 3. SFG Synthesis of General High-Order All-Pass Current Transfer Functions

### 3.1. Realization Procedure

The general form of an *n*th-order all-pass current transfer function can be expressed by the following expression:

(3) Io(s)Iin(s) =(−1)nbnsn+(−1)n−1bn−1sn−1+⋯+b2s2−b1s+1bnsn+bn−1sn−1+⋯+b2s2+b1s+1.

**Figure 3:**
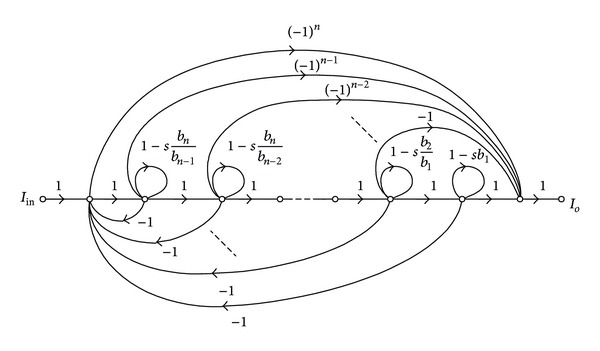
SFG corresponding to (3).

**Figure 4:**
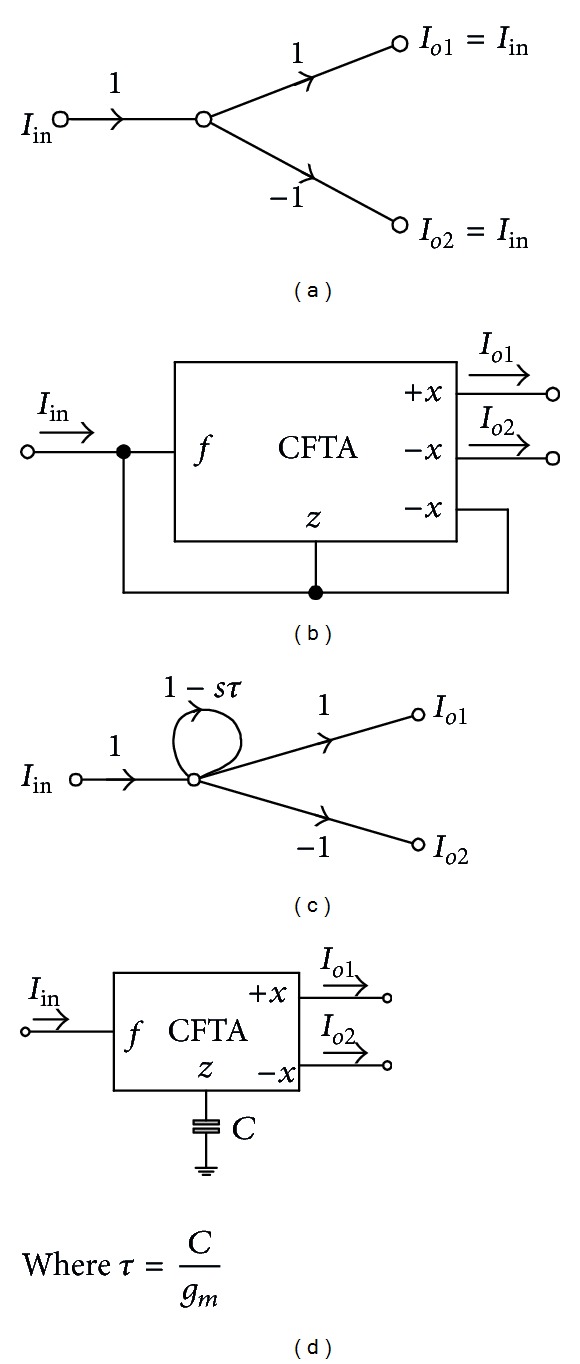
Subgraphs of Figure 3 and their corresponding active-*C* subcircuit involving CFTAs.

**Figure 5:**
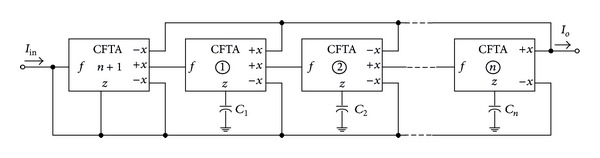
CFTA-based realization of *n*th-order current-mode all-pass filter, corresponding to the SFG given in Figure 3.

Equation (3) can be represented by the SFG as shown in Figure 3. It is clearly seen that the graph consists of two basic operations, which are multioutput current follower and current lossless integrator, as redrawn in Figures 4(a) and 4(c), respectively. Using the current and voltage relations of the CFTA given in (1), we easily find that these two subgraphs can be realized using CFTA by the subcircuits as shown in Figures 4(b) and 4(d), respectively. For the CFTA-based realization, it can readily obtain the CFTA-C circuit by interconnecting the corresponding subcircuits of Figures 4(b) and 4(d) according to the overall signal flow graph representation of Figure 3. Therefore, the CFTA-C circuit realizing any *n*th-order all-pass current transfer function can be shown in Figure 5. For this realization, it has to be noted that, for general *n*th-order filter function, the proposed filter configuration requires *n* + 1 CFTAs as active elements and *n* capacitors as passive elements. Also note that the circuit realization uses only grounded capacitors that are suitable for the integrated circuit implementation point of view and also exhibits low-input impedance and high-output impedance terminals that are desirable for cascading in current-mode operation [17].

From the CFTA-based circuit realization, the design equations can be obtained through comparing the SFG representation of Figure 3 with Figure 5. The results are summarized as follows:

(4) bnbn−1=C1gm1bn−1bn−2=C2gm2   ⋮  b2b1=Cn−1gm(n−1),b1=Cngm(n).

It should be noted from the above expressions that the coefficients *b*_*i*_  (*i* = 1,2,…, *n*) of the realized function can be tuned electronically by adjusting the *g*_*m*_-value of the CFTA.

### 3.2. Nonideal Analysis

In this subsection, the effect of the non-idealities of the CFTA on the circuit performance is discussed. In case of the nonideal characteristic condition, the port relations of the CFTA given in (1) can be rewritten as follows:

(5) $$\begin{array}{c} v_{f}=0,\;\;i_{z}=\alpha i_{f},\;\;i_{x}=\beta g_{m}Z_{z}i_{z}, \end{array}$$

where *α*  is the nonideal current gain from *f* to *z* terminals, and *β* is transconductance inaccuracy factor from *z* to *x* terminals of the CFTA, respectively. Reanalyzing the realized structure in Figure 5 with (5) yields the following nonideal current transfer function:

(6) Io(s)Iin(s) =γ[(−1)nbnsn+(−1)n−1bn−1sn−1+⋯+b2s2−b1s+1bnsn+γbn−1sn−1+⋯+γb2s2+γb1s+γ],

where

(7) bnbn−1=C1α1β1gm1bn−1bn−2=C2α2β2gm2   ⋮ b2b1=Cn−1αn−1βn−1gm(n−1) b1=Cnαnβngm(n),γ=αn+1βn+1gm(n+1)rz(n+1)1−αn+1+[αn+1βn+1gm(n+1)rz(n+1)].

In (7), *α*_*i*_ and *β*_*i*_ (for *i* = 1,2,…, *n*) represent the parameters *α* and *β* of the *i*th-CFTA, respectively. Practically, these transfer gains differ from unity by tracking errors of the CFTA. More specifically, *r*_*z*(*n*+1)_ denotes the parasitic resistance at the *z*-terminal of the (*n* + 1)th-CFTA, which is ideally equal to infinity.

### 3.3. Design Example

To demonstrate the proposed design procedure, the third-order all-pass transfer function is considered. Generally, the current transfer function of the normalized third-order all-pass filter is defined as follow:

(8) Io(s)Iin(s)=−b3s3+b2s2−b1s+1b3s3+b2s2+b1s+1=−s3+2s2−2s+1s3+2s2+2s+1.

**Figure 6:**
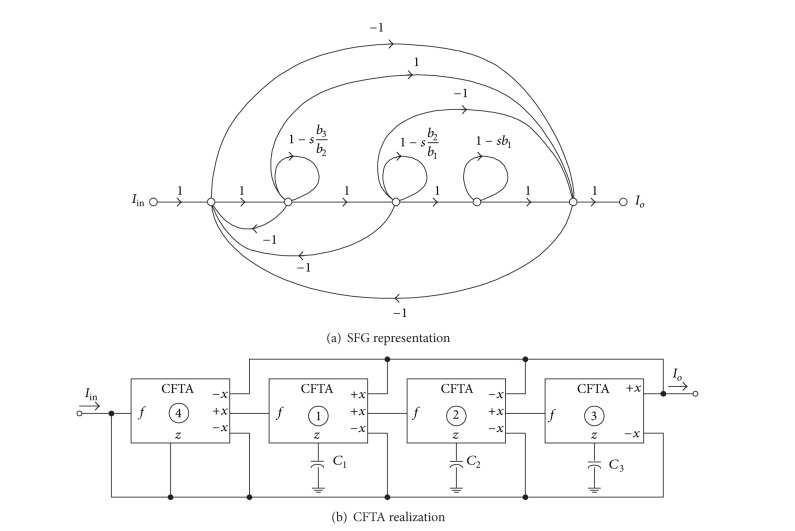
Third-order all-pass current transfer function of (10).

It can be easily shown that the above transfer function can be represented by the SFG shown in Figure 6(a), and the corresponding circuit realization of this graph is thus shown in Figure 6(b). In this example, the design equations of the circuit are found as follow:

(9) b3b2=C1gm1=12,  b2b1=C2gm2=1,  b1=C3gm3=2.

Thus, the normalized component values are obtained as *C*_1_ = *C*_2_ = *C*_3_ = 1 F, *g*_*m*1_ = 2 A/V, *g*_*m*2_ = 1 A/V, and *g*_*m*3_ = 1/2 A/V. Routine circuit analysis of Figure 6(b) yields the current transfer function as follows:

(10) Io(s)Iin(s)=−s3+(gm1C1)s2−(gm1gm2C1C2)s+(gm1gm2gm3C1C2C3)×(s3+(gm1C1)s2+(gm1gm2C1C2)s  +(gm1gm2gm3C1C2C3))−1.

The active and passive sensitivities of various parameters for the designed circuit of Figure 6(b) are analyzed and the results are given in Table 1. It is clearly seen that all the sensitivities are low and within unity in magnitude.

## 4. SFG Synthesis of General High-Order All-Pole Current Transfer Functions

**Figure 7:**
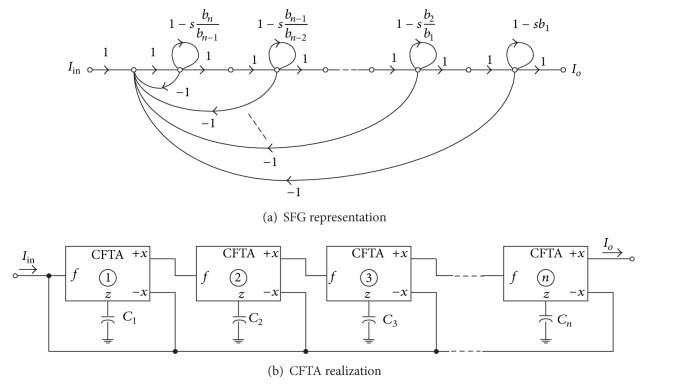
Realization of *n*th-order all-pole lowpass current transfer function.

Considering the SFG of Figure 3 if all the forward parts are removed, we obtain the suitable SFG as redrawn in Figure 7(a). In this case, the corresponding current transfer function is expressed in the following form:

(11) $$\begin{array}{c} \frac{I_{o}\left(s\right)}{I_{\text{in}}\left(s\right)}=\frac{1}{b_{n}s^{n}+b_{n-1}s^{n-1}+\cdots +b_{1}s+1}. \end{array}$$

It is apparent that this equation is now in the form of general *n*th-order all-pole lowpass current transfer function. Since the SFG representation of this function is slightly modified from Figure 3, then the CFTA-based circuit realization can easily be obtained by using the same realization procedure presented in Figure 4. The resulting circuit is shown in Figure 7(b). It is clearly seen that the circuit includes at most *n* CFTAs and *n* grounded capacitors. Furthermore, the design equations of the circuit in Figure 7(b) are also the same as given in (4).

As an example of the useful application of the given second structure, let us consider the third-order all-pole lowpass Butterworth function. In this case, the current transfer function has the canonical form:

(12) $$\begin{array}{c} \frac{I_{o}\left(s\right)}{I_{\text{in}}\left(s\right)}=\frac{1}{b_{3}s^{3}+b_{2}s^{2}+b_{1}s+1}=\frac{1}{s^{3}+2s^{2}+2s+1}. \end{array}$$

**Figure 8:**
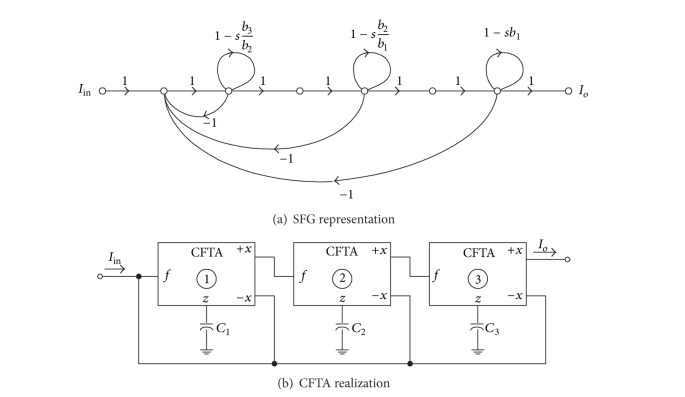
Third-order all-pole lowpass Butterworth current transfer function.

The SFG representing (12) can be drawn in Figure 8(a). Referring to Figure 4, the circuit realization of this graph is shown in Figure 8(b) and has the following:

(13) $$\begin{array}{c} \frac{b_{3}}{b_{2}}=\frac{C_{1}}{g_{m1}}=\frac{1}{2},\;\;\frac{b_{2}}{b_{1}}=\frac{C_{2}}{g_{m2}}=1,\;\;b_{1}=\frac{C_{3}}{g_{m3}}=2. \end{array}$$

Thus, the normalized component values are obtained as  *C*_1_ = *C*_2_ = *C*_3_ = 1 F,  *g*_*m*1_ = 2 A/V, *g*_*m*2_ = 1 A/V, and *g*_*m*3_ = 1/2 A/V. Routine circuit analysis of Figure 8(b) yields the current transfer function as follows:

(14) Io(s)Iin(s)=(gm1gm2gm3C1C2C3)×(s3+(gm1C1)s2+(gm1gm2C1C2)s  +(gm1gm2gm3C1C2C3))−1.

From (14), the coefficient sensitivities to active and passive components (|*S*_*g*_*mi*__^*b*_*i*_^| and |*S*_*C*_*i*__^*b*_*i*_^|) are 1 or 0. Also, the active and passive sensitivities of the natural angular frequency and quality factor (|*S*_*g*_*mi*_,*C*_*i*__^*ω*_*o*_^| and |*S*_*g*_*mi*_,*C*_*i*__^*Q*^|) are calculated and found as 1 or 0. Thus, all the sensitivities are small.

## 5. Computer Simulations and Results

To verify the theoretical analysis, the proposed design procedure given above has been simulated with PSPICE simulation program. To implement the CFTA active device in simulations, the bipolar technology structure depicted in Figure 2 has been employed using transistor model parameters PR100N (PNP) and NP100N (NPN) of the bipolar arrays ALA400. The DC supply voltages and bias currents were set as +*V* = −*V* = 2 *V* and *I*_*B*_ = 100 *μ*A, respectively.

**Figure 9:**
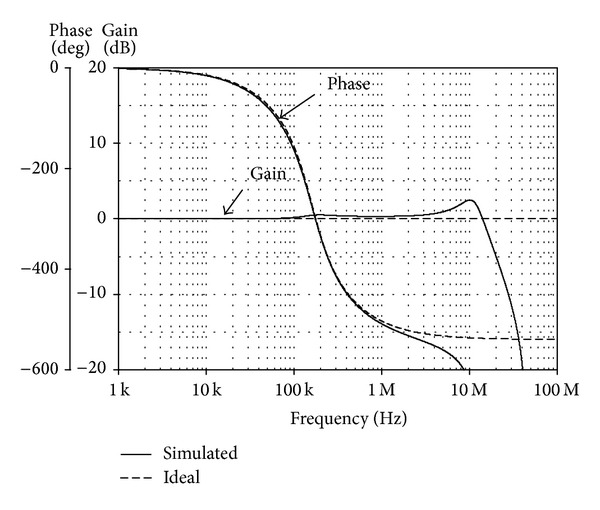
Simulated and ideal frequency responses of the realized third-order all-pass circuit in Figure 6(b).

**Figure 10:**
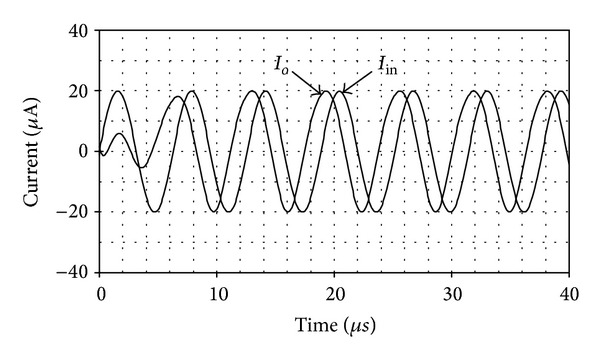
Time-domain responses of the realized third-order all-pass circuit in Figure 6(b).

**Figure 11:**
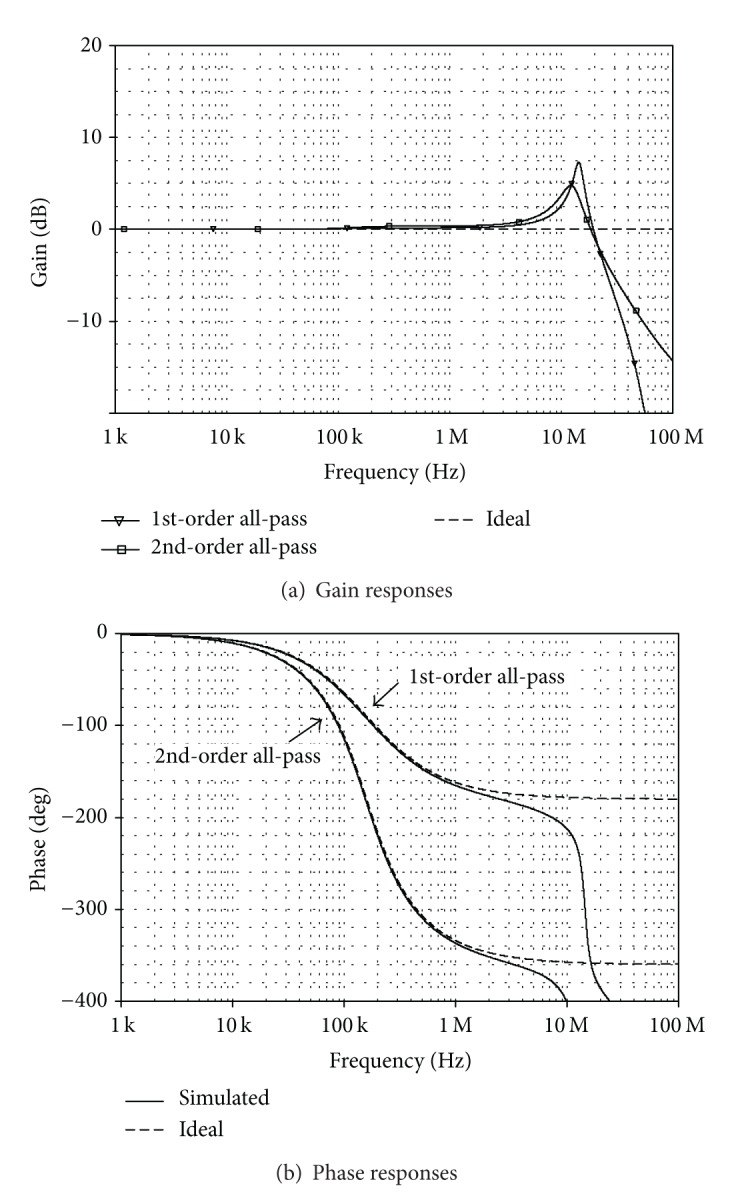
Simulated and ideal frequency responses of the current-mode all-pass circuit in Figure 5 when *n* = 1, 2.

As an example, the illustrative current-mode third-order all-pass filter of Figure 6(b) was designed with *ω*_*o*_ = 10^6^ rad/sec. For this purpose, the denormalized component values were chosen as *C*_1_ = *C*_2_ = *C*_3_ = 1 nF, *g*_*m*1_ = 2 mA/V (*I*_*o*1_≅100 *μ*A), *g*_*m*2_ = 1 mA/V (*I*_*o*2_≅50 *μ*A), *g*_*m*3_ = 1/2 mA/V (*I*_*o*3_≅25 *μ*A), and  *g*_*m*4_ = 1 mA/V (*I*_*o*4_≅50 *μ*A). The simulated frequency responses comparing with the theoretical values are shown in Figure 9. We see that the phase response is found to vary with frequency from 0° to −540°, with a value of −270° at the pole frequency *f*_*o*_≅159 kHz. From the results, the simulated *f*_*o*_ was found to be 154 kHz, which is in close proximity to the ideal value. Next, applying a sinusoidal current input signal of 20 *μ*A peak at 159 kHz to the circuit, the input and output waveforms are given in Figure 10. This results in 4.86 *μ*s time delay at the output current corresponding to −278° phase shift. In Figure 11, the frequency characteristics of the CFTA-based current-mode all-pass filter in Figure 5 for *n* = 1 and 2 are also depicted. It can be observed that the simulation results agree very well with the ideal ones.

**Figure 12:**
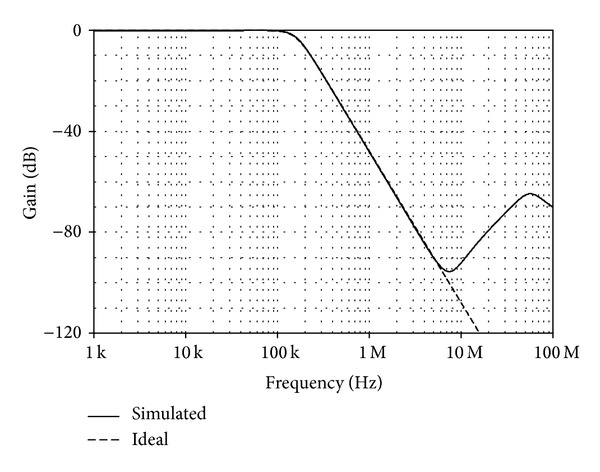
Simulated and ideal third-order all-pole lowpass Butterworth current responses of Figure 8(b).

For the CFTA-based third-order all-pole lowpass Butterworth circuit realization of Figure 8(b), the circuit with the pole frequency of *f*_*o*_≅159 kHz was designed with the following settings: *C*_1_ = *C*_2_ = *C*_3_ = 1 nF, *g*_*m*1_ = 2 mA/V, *g*_*m*2_ = 1 mA/V and *g*_*m*3_ = 1/2 mA/V. The simulated results and ideal values of the gain responses are depicted in Figure 12.

## 6. Conclusions

This study presents a signal flow graph (SFG) approach to synthesize the general, high-order, all-pass, and all-pole lowpass current transfer functions by an active-*C* circuit. By using the SFG representation, any *n*th-order all-pass current transfer function can be realized by employing *n* + 1 CFTAs and *n* grounded capacitors, and *n*th-order all-pole lowpass current transfer function can also be realized employing *n* CFTAs and *n* grounded capacitors. It has been shown that the design procedure given here is resistorless structure, electronic tuning property, and suitable for integration. Furthermore, the circuit has low sensitivity. PSPICE simulation results which agree very well with the theoretical analysis are also included.

## Figures and Tables

**Table 1:** Sensitivities for the circuit parameters in Figure 6(b).

	*g*_*m*1_	*g*_*m*2_	*g*_*m*3_	*C*_1_	*C*_2_	*C*_3_
b_0_	1	1	1	−1	−1	−1
b_1_	1	1	0	−1	−1	0
b_2_	1	0	0	−1	0	0
ω_0_	0	1	0	0	−1	0
